# Transcutaneous electrical nerve stimulation (TENS): towards the development of a clinic-friendly method for the evaluation of excitatory and inhibitory pain mechanisms

**DOI:** 10.1080/24740527.2020.1862624

**Published:** 2021-03-23

**Authors:** Monica Sean, Alexia Coulombe-Lévêque, Matthieu Vincenot, Marylie Martel, Louis Gendron, Serge Marchand, Guillaume Léonard

**Affiliations:** a Research Centre on Aging, Sherbrooke, Quebec, Canada; b School of Rehabilitation, Faculty of Medicine and Health Sciences, Université de Sherbrooke, Sherbrooke, Quebec, Canada; c Centre de recherche du CHUS, Sherbrooke, Quebec, Canada; d Department of Pharmacology-Physiology, Faculty of Medicine and Health Sciences, Université de Sherbrooke, Sherbrooke, Quebec, Canada; e Department of Neurosurgery, Faculty of Medicine and Health Sciences, Université de Sherbrooke, Sherbrooke, Quebec, Canada

**Keywords:** pain, transcutaneous electrical nerve stimulation (TENS), conditioned pain modulation, temporal summation, endogenous pain modulation

## Abstract

**Background**: Temporal summation and conditioned pain modulation (CPM) can be measured using a thermode and cold pressor test (CPTest). Unfortunately, these complex and expensive tools are ill-suited for routine clinical assessments.

**Aims**: We aimed to compare the temporal summation and CPM obtained with the thermode + CPTest paradigm to those obtained with a novel paradigm using transcutaneous electrical nerve stimulation (TENS).

**Methods**: We assessed temporal summation and CPM in 29 healthy participants, using two paradigms (random order): TENS, and thermode + CPTest. In the TENS paradigm, both the conditioning stimulus (CS) and the test stimulus (TS) were delivered using TENS; in the thermode + CPTest paradigm, the CS consisted of a CPTest and the TS was delivered using a thermode. We compared the average temporal summation and CPM evoked by the two paradigms.

**Results**: Average temporal summation was similar for both modalities (*P* = 0.90), and the number of participants showing temporal summation was similar in both paradigms (19 with thermode vs. 18 with TENS; *P* = 1.00). Average CPM response was larger following the thermode + CPTest than following the TENS (*P* = 0.005), and more participants showed CPM with the thermode + CPTest paradigm compared to the TENS paradigm (24 vs. 14; *P* = 0.01).

**Conclusions**: Both paradigms were roughly equivalent in the ability to evoke temporal summation (although response to one modality did not predict response to the other), but the TENS paradigm appeared to be less apt to induce a CPM response than the thermode + CPTest paradigm.

## RÉSUMÉ

**Contexte**: La sommation temporelle et la modulation de la douleur conditionnée (MDC) peuvent être mesurées à l’aide d’une thermode et d’un test au froid. Malheureusement, ces tests complexes et coûteux sont mal adaptés aux évaluations cliniques de routine.

**Objectifs**: Nous avons cherché à comparer la sommation temporelle et la modulation de la douleur conditionnée obtenues avec le paradigme thermode + test au froid à ceux obtenus avec un nouveau paradigme utilisant la neurostimulation électrique transcutanée (TENS).

**Méthodes**: Nous avons évalué la sommation temporelle et la modulation de la douleur conditionnée chez 29 participants en bonne santé, en utilisant les deux paradigmes (ordre aléatoire) : TENS, et thermode + test au froid. Dans le paradigme TENS, Le stimulus de conditionnement et le stimulus d’essai ont été transmis à l’aide de la neurostimulation électrique transcutanée ; dans le paradigme thermode + test au froid, le stimulus de conditionnement consistait en un test au froid et le stimulus d’essai était transmis à l’aide d’une thermode. Nous avons comparé la sommation temporelle et la modulation de la douleur conditionnée moyennes évoqués par les deux paradigmes.

**Résultats**: La sommation temporelle moyenne était similaire pour les deux modalités (P = 0,90), et le nombre de participants ayant montré une sommation temporelle étaient similaires dans les deux paradigmes (19 avec la thermode contre 18 avec la TENS; P = 1,00). La réponse moyenne de modulation de la douleur conditionnée était plus importante après la thermode + test au froid qu’après la neurostimulation électrique transcutanée (P = 0,005), et un plus grand nombre de participants ont montré une modulation de la douleur conditionnée avec la thermode + test au froid par rapport au paradigme TENS (24 contre 14 ; P = 0,01).

**Conclusions**: Les deux paradigmes sont à peu près équivalents en ce qui concerne la capacité d’évoquer la sommation temporelle (bien que la réaction à une modalité ne prévoie pas la réaction à l’autre), mais le paradigme TENS semble moins apte à induire une réponse de modulation de la douleur conditionnée que le paradigme thermode + test au froid.

## Introduction

Chronic pain affects approximately one-fifth of the general population, including one-third of the elderly population.^1^ A number of chronic pain conditions are characterized by imbalances in endogenous excitatory and/or inhibitory mechanisms of pain modulation.^2–5^ Animal studies have identified the specific electro-neurophysiological correlates of some of these mechanisms, such as wind-up (an excitatory mechanism of pain characterized by a progressive increase in dorsal horn activity observed following repeated C-fiber activation)^6,7^ and diffuse noxious inhibitory controls (an endogenous inhibitory mechanism of pain characterized by the activation of descending inhibitory projections from brainstem structures following a noxious stimulation, resulting in decreased reponses of spinal cord neurons).^8,9^ Human studies, where such electro-neurophysiological measurements are much more difficult to obtain, often rely on behavioral correlates of these endogenous pain control mechanisms: for excitatory mechanisms, a phenomenon called “temporal summation” is often measured, wherein perceived pain intensity increases throughout a repetitive noxious stimulation of fixed intensity^10,11^; for inhibitory mechanisms, the concept of conditioned pain modulation (CPM) was developed, wherein a noxious stimulation induces widespread hypoalgesia.^12,13^

Imbalances in these endogenous pain modulation mechanisms (often in the form of increased temporal summation and/or decreased CPM, which both result in greater overall pain intensity)^14,15^ appear to correlate with response to certain treatments.^16–18^ For instance, patients with chronic pancreatitis presenting increased temporal summation respond preferentially to pregabalin,^16^ and patients with diabetic neuropathy presenting decreased CPM respond preferentially to duloxetine.^18^ Evaluating these mechanisms among people suffering from chronic pain could therefore allow clinicians to adapt the treatment to each patient according to the deficits observed.^18–21^ A paradigm often used to assess CPM consists of a test stimulus (TS) administered before and after a conditioning stimulus (CS); CPM is measured by calculating the difference in pain levels elicited by the TS before and after the CS.^22^ Our team has developed a slightly modified version of this paradigm that allows for the assessment of temporal summation as well as CPM, by measuring temporal summation throughout the first TS.^23,24^ In this modified CPM paradigm, the TS consists of a sustained moderately painful heat stimulation delivered for 120 s using a thermode and the CS consists of a cold pressor test (CPTest) wherein subjects immerse their dominant forearm in a cold water bath (10°C) for 120 s. The continuous TS stimulation was chosen (as opposed to pulsed stimulations^25,26^; see Discussion) because our team has developed a specific expertise with this particular method. Unfortunately, although this thermode + CPTest paradigm is well suited to the laboratory setting, it is relatively time-consuming and requires complex and costly equipment^27^; as such, it is not realistic for clinicians to use it for routine patient assessment.

A potential alternative tool to study excitatory and inhibitory mechanisms is transcutaneous electrical nerve stimulation (TENS). TENS, which is frequently used in rehabilitation as a hypoalgesic modality,^28–31^ is a noninvasive tool that stimulates A-delta and C fibers: depending on the parameters, it can either block pain signals (high-frequency [50–100 Hz], low-intensity mode) or elicit a painful sensation that will trigger inhibitory mechanisms (low-frequency [1–5 Hz], high-intensity mode).^19,32^ Here, we hypothesized that TENS, when used in low-frequency and high-intensity mode, provokes a painful sensation that could potentially be used as both the TS and the CS in the CPM paradigm, replacing the thermode and CPTest. Unlike these tools, TENS is affordable, is easy to use, and requires very little time and equipment.

The objective of this exploratory study was to determine whether TENS can be used to evaluate temporal summation and CPM in healthy participants. More specifically, we aimed to compare the average temporal summation and CPM scores obtained with the two paradigms, to determine whether the two paradigms triggered these pain mechanisms in a similar number of participants and to assess whether response to one paradigm correlates with response to the other.

## Methods

This study was conducted according to the Declaration of Helsinki. All participants provided written informed consent for their participation in this study. Ethics approval was granted from the Comité éthique de recherche (CÉR) du CIUSSS de l’Estrie–CHUS, Sherbrooke, Quebec, Canada (2019-3022).

### Participants

Participants were eligible to take part in our study if they were at least 18 years of age, and for women, if they were in their preovulatory phase, had undergone menopause, or were using a hormonal contraceptive (oral contraceptive, intrauterine device, etc.).^33^ Participants were excluded if they suffered from chronic pain, neurological disorders, or musculoskeletal disorders; if they had history of nonefficacy with TENS; or if they presented contraindications related to TENS (history of epilepsy, presence of a pacemaker, or metal implants^34^).

Participants were asked to refrain from taking analgesics during the 7 days preceding each experimental session and to refrain from consuming caffeine and from doing intense physical activity during the 24 h preceding each experimental session.

### Study Design

Study participants attended two experimental sessions at the Research Centre on Aging of the CIUSSS de l’Estrie–CHUS, during which their temporal summation and CPM response were assessed. In one session, this assessment was done using TENS; in the other, using a thermode and CPTest. Session order was randomized between participants (randomization by block of four stratified by sex, using a random number table). The two sessions were separated by 24 to 72 h and lasted approximately 1 h each. One experimenter (female physiotherapy student) oversaw the TENS procedure. The other experimenter (female pharmacology student) oversaw the thermode/cold pressor procedure. Both experimenters used standardized techniques and procedures.

### Testing Sequence and Apparatus

#### Thermode and CPTest

**Figure 1.**
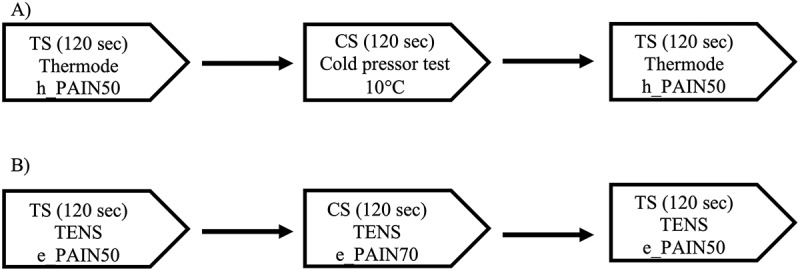
Testing sequence of the thermode + CPTest paradigm (A) and the TENS paradigm (B)

We used a CPM testing procedure^23^ consisting of a TS administered before and after a CS. The TS was generated by a 3 cm × 3 cm thermode (TSA II, NeuroSensory Analyzer, Medoc Instruments, Durham, NC) applied on the nondominant forearm of participants. Pain perception was continuously recorded with a computerized visual analog scale (CoVAS) linked to the thermode. The CoVAS consists of a mechanical slider running along a 10-cm horizontal track housed in a box connected to a computer. Participants moved the slider to reflect their pain, with the left track boundary identified as “no pain” (score = 0) and the right track boundary identified as “intolerable pain” (score = 100). Participants were asked to continuously rate their pain levels by moving the slider on the device. The CoVAS sampling rate was set at 10 Hz. Figure 1A summarizes the testing sequence for the thermode + CPTest paradigm.

#### TENS

Unless otherwise specified, the TENS procedure was identical to the thermode + CPTest procedure outlined above. The TS was generated by a TENS Eclipse Plus digital apparatus (Empi, St. Paul, MN) with carbon electrodes (4 × 4 cm). TENS parameters were set as per low-frequency, high-intensity mode (2 Hz, 400 µs). Pain was assessed with a 10 cm VAS with the left boundary identified as “no pain” (score = 0) and the right boundary identified as “intolerable pain” (score = 100). The VAS was on paper and participants were asked to rate their pain perception by tracing a line on the VAS with a pencil. The VAS was preferred to the CoVAS because it is much cheaper and better suited to the daily reality of clinical practice. Pain measurements were taken at 15, 30, 60, 90, and 120 s. Figure 1B summarizes the testing sequence for the TENS paradigm.

### Practice Test and pretests

#### Thermode and CPTest Paradigm

For the practice test and pretests with the thermode, temperature was gradually increased from 32°C to a maximum of 51°C at a rate of 0.3°C/s. One practice test was performed on the nondominant anterior forearm of each participant so that they could become familiar with the apparatus and nociceptive stimulus (heat stimulation). Participants were asked to verbally identify the point at which the heat became painful (pain perception threshold) and the point at which the pain was no longer tolerable (pain tolerance threshold). For pretests, the thermode was placed on the nondominant anterior forearm of each participant, and pain was rated using the CoVAS. Participants were advised that the cursor should remain at the left boundary while they felt no pain; that they should start moving it toward the right when the heat became painful; and that the cursor should be at the right boundary when their pain was no longer tolerable.^23^ Pretests were repeated until subjects’ pain reports were consistent (within 1°C) between trials. Pretest results were used to identify the h_PAIN50 temperature for each participant, defined as the temperature eliciting a pain of 50/100. The h_PAIN50 temperature was used as the formal TS.

#### TENS Paradigm

For the practice test and pretests with TENS, the electrodes were placed over the sural nerve of the nondominant ankle and current was gradually increased from 0 mA to a maximum of 60 mA at a rate of 1 mA/s. The sural nerve was chosen instead of the forearm (which would have been more similar to the thermode + CPTest paradigm) because it is a pure sensory nerve that is not involved in voluntary muscle contraction (and the chosen location is not adjacent to muscle mass), such that its stimulation does not induce unpleasant and/or distracting muscle contractions. For pretests, participants verbally identified the point at which the electrical stimulation elicited a pain level of 50/100 e_PAIN50). Pretests were conducted twice and the average e_PAIN50 current was used as the formal TS. A second series of pretests was conducted on the dominant ankle, this time to identify the e_PAIN70 (current intensity at which the stimulation elicited a pain level of 70/100), which was used as the formal CS.

### Test Stimulus

#### Thermode and CPTest Paradigm

The TS in this paradigm consisted of a noxious heat stimulus generated by the thermode, applied for 2 min on the nondominant anterior forearm at the predetermined, individually tailored h_PAIN50 temperature.^23,24^ Subjects were told that the thermode temperature could increase, remain stable, or decrease over the course of the stimulation, and they were instructed to continuously record their pain level using the CoVAS. In fact, after a constant rise (0.3°C/s) from baseline (32°C) to the predetermined temperature, the temperature remained fixed throughout the test (120 s total). The TS was administered twice: before the CS and after the CS. During each TS, participants were asked to rate their level of pain. The pre-CS TS served two purposes: it provided a way to evaluate temporal summation, and it served as the baseline against which post-CS TS scores were compared to assess CPM efficacy.

#### TENS Paradigm

The TS in the TENS paradigm, which served the same purpose as the thermode used in the thermode + CPTest paradigm, consisted of a noxious electrical stimulus generated by TENS, applied for 120 s over the sural nerve of the nondominant ankle at the predetermined, individually tailored e_PAIN50 current amplitude. Participants were asked to rate their pain level at *t* = 15, 30, 60, 90, and 120 s by tracing a line on a pain VAS. A new scale was provided at each sampling time so that participants did not see their previous line.

### Conditioning Stimulus

#### Thermode and CPTest Paradigm

The CS in this paradigm consisted of a CPTest, wherein participants immersed their dominant forearm in a cold water bath for 120 s. During the CPTest, patients verbally rated the intensity and unpleasantness of their pain on a 100-point numerical pain scale (with the same anchors as the VAS) at *t* = 15, 30, 60, 90, and 120 s. These scores were used to calculate the average pain evoked by the CS to ensure that the two paradigms were comparable in terms of the average pain intensity evoked by the CS.

#### TENS Paradigm

The CS in the TENS paradigm consisted of a noxious electrical stimulus generated by TENS, applied for 120 s on the dominant ankle (sural nerve) at the predetermined, individually tailored e_PAIN70 current amplitude. Participants rated their pain as in the thermode + CPTest paradigm.

### Outcome Measures: Temporal Summation

The efficacy of excitatory mechanisms was measured by evaluating pain fluctuations (i.e., temporal summation) during the pre-CS TS.^23^ Temporal summation can be measured in various ways, although most have important drawbacks (e.g., subtracting the pain score at 30 s from the pain score at 120 s^23^ depends on two arbitrarily chosen points at the expense of the general trend; subtracting the lowest pain score from the highest pain score^35^ risks misattributing early, nonsustained fluctuations in pain scores to temporal summation). After careful consideration, we elected to measure temporal summation as a linear regression obtained from the pain scores at *t* = 30, 60, 90, and 120 s. The slope of that linear regression was used as a measure of temporal summation, such that positive scores represented *activation* of excitatory mechanisms. Participants with a slope greater than or equal to 0.1 (corresponding to an increase of roughly 10 percentage points in pain scores) were considered to show temporal summation.

### Outcome Measures: CPM

To facilitate comparisons for pain inhibitory mechanisms, pain intensity ratings obtained during the 120-s TS were averaged, and this mean was used in subsequent analyses. The magnitude of CPM was obtained by subtracting pre-CS TS pain scores from post-CS TS pain scores, such that negative values represented activation of inhibitory mechanisms.^23^

### Statistical Analysis

Normality was assessed using Shapiro-Wilk tests and visual inspection of the data. Because normality could not be assumed, nonparametric tests were used. Temporal summation scores were averaged across participants within each paradigm and compared between paradigms using Wilcoxon’s signed-rank test. The same analysis was conducted for CPM scores. Other average comparisons (e.g., average CS intensity) were also done using Wilcoxon’s signed-rank test. The correlation between temporal summation scores obtained from the two paradigms was assessed with Spearman analysis. Again, the same analysis was done for CPM scores. Temporal summation scores (continuous) were then transformed into a dichotomic “temporal summation present/absent” score, wherein temporal summation was considered “present” if the linear regression obtained from pain scores had a slope of 0.1 or more (corresponding roughly to a pain increase of 10 percentage points). McNemar’s test was carried out to identify whether more participants showed temporal summation in one paradigm compared to the other, and the relationship between the dichotomic temporal summation scores obtained with the two paradigms was assessed with the φ coefficient (mean square contingency coefficient). Specifically, the φ coefficient was calculated to assess the association between a participant’s response to both paradigms (i.e., to determine whether temporal summation was consistently evoked [or not] by the two modalities). CPM scores (continuous) were similarly transformed into dichotomic scores (CPM present/absent, wherein CPM was considered present when the delta pain score was less than or equal to −10, corresponding to a reduction of pain of at least 10 percentage points) and the same analyses were carried out.

The threshold of 10 percentage points was chosen for these transformations into dichotomic scores because fluctuations in pain scores less than 10 percentage points were considered random fluctuations and not representative of an actual change in pain perception.

In an effort to increase reproducibility and reduce the rate of false positives reported in the literature and to promote scientific rigor within the field, we embrace the changes proposed by Benjamin et al., wherein the threshold for statistical significance is lowered to *P* < 0.005 and *P* values ranging between 0.05 and 0.005 are instead considered “suggestive.”^36^ Descriptive statistics are presented as means ± standard deviations in the text and as means ± standard errors in the figures.

## Results

### Participants and Baseline Data

Forty-six participants were interested in taking part in our study, but 11 of them did not meet eligibility criteria, so 35 participants were invited to our experimental sessions. Six of these participants were later excluded for various reasons: failure to attend the second session; mild adverse reaction to TENS; no pain felt during TENS; history of nonefficacy with TENS; inability to withstand the CPTest for more than 15 s; and undisclosed neurocognitive disorder. The 29 remaining participants (14 men and 15 women) were aged 35 ± 18 years old (range: 21–71 years old). All participants complied with the instructions relating to medication, caffeine use, and physical activity prior to the experimental sessions.

The average thermode temperature used for the TS was 46.7°C ± 2.0°C, and the average TENS current was 37 ± 10 mA. Despite our pretests, our results suggest that the average pain intensity induced during the pre-CS TS was higher with the thermode than with the TENS (54 ± 16/100 and 39 ± 21/100, respectively; *P* = 0.007). The average TENS current used for the CS was 38 ± 9 mA. The intensity of pain during the CS was 55 ± 26/100 with the CPTest and 46 ± 26/100 with TENS (*P* = 0.06), and the unpleasantness of pain evoked by the CS was 67 ± 23/100 with the CPTest and 56 ± 25/100 with the TENS (*P* = 0.06).

### Temporal Summation

**Figure 2.**
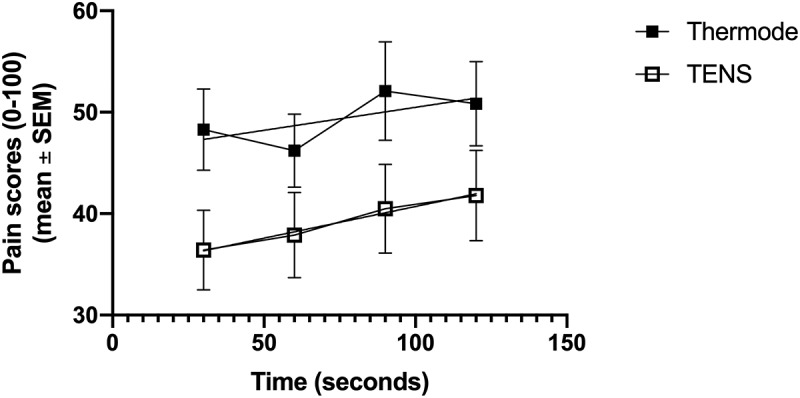
Pain scores evoked by the TS before and after the CS, for both conditions

Temporal summation, calculated as the slope of the linear regression obtained from the pain scores during the pre-CS TS (at *t* = 30, 60, 90, and 120 s), was similar for both modalities (0.05 ± 0.30 with the thermode and 0.06 ± 0.16 with the TENS; *P* = 0.90; see Figure 2).There was no correlation (*r* = −0.26; *P* = 0.17) between the temporal summation (continuous variable) induced by the two modalities. To assess the presence or absence of temporal summation induced by the thermode and TENS for each participant, temporal summation was transformed from a continuous variable (slope) into a dichotomic variable (present/absent; see Methods). This allowed us to determine whether both paradigms (or only one, or neither) evoked temporal summation in each participant (Table 1). Table 1 shows the response (pain mechanism present/absent) of each participant to the two paradigms (TENS vs. thermode + CPTest). For each pain mechanism (temporal summation (left column) and CPM (right column)), we assessed whether a participant’s response was the same with both modalities, or whether they responded to only one modality. Inconsistent responses (i.e., presence of temporal summation/CPM with only one of the two modalities) are highlighted. McNemar’s test was used to determine whether more participants showed temporal summation (left) and CPM (right) in response to one modality compared to the other. The φ coefficient (mean square contingency coefficient) was calculated to assess the association between participants’ response to the two modalities, for each pain mechanism (temporal summation (left) and CPM (right) (i.e., whether a given pain mechanism is consistently evoked by the two modalities). The number of participants showing temporal summation was similar in both paradigms (19 with thermode vs. 18 with TENS; *P* = 1.00). However, there was no association between participants’ responses to the two modalities (in other words, response to one modality was not associated with response to the other modality; φ = −0.27; *P* = 0.15). Table 2A summarizes temporal summation responses obtained with the two paradigms (thermode + CPTest vs. TENS).

**Table 1.** Individual CPM and TSP response to the two paradigms

Participant		Temporal summation	CPM
P1		Thermode	Thermode
P2		None	Thermode
P3		Both	None
P4		None	Thermode
P5		TENS	None
P6		None	None
P7		TENS	TENS
P8		None	None
P9		TENS	None
P10		Thermode	Thermode
P11		Thermode	None
P12		TENS	None
P13		None	Thermode
P14		Thermode	None
P15		TENS	None
P16		Thermode	Both
P17		TENS	None
P18		None	TENS
P19		Thermode	Thermode
P20		TENS	Both
P21		Thermode	Thermode
P22		None	Thermode
P23		Thermode	None
P24		TENS	Thermode
P25		Both	None
P26		Thermode	Thermode
P27		None	Thermode
P28		None	Both
P29		None	Thermode
Same response to both modalities	Present with both modalities	10	12
	Absent with both modalities	2	3
Different response to the two modalities	Presence with thermode only	9	12
	Presence with TENS only	8	2
Statistical analysis	*McNemar’s p*	*p* = 1.00	*p* = 0.01*
	*Phi coefficient* (φ)	ϕ = −0.27; *P* = 0.15	ϕ = 0.08; *P* = 0.68

*Statistically suggestive difference. **Statistically significant difference.

**Table 2.** Summarizes temporal summation (2A) and CPM (2B) responses obtained with the two paradigms (thermode + CPTest vs TENS)

A)			
Temporal summation			
	Thermode + CPTest	TENS	
Average score (slope of linear regression)	0.05 ± 0.3	0.06 ± 0.16	*p* = 0.9
			*r* = −0.26; *p* = 0.17
Participants showing temporal summation (*n*)	19	18	*p* = 1.00
			ϕ = −0.27; *p* = 0.15
B)			
CPM			
	Thermode + CPTest	TENS	
Average score (delta pain score)	−11 ± 20	−1 ± 12	*p* = 0.005**
			*r* = −0.29; *p* = 0.13
Participants showing CPM (*n*)	24	14	*p* = 0.01*
			ϕ = −0.08; *p* = 0.68

*Statistically suggestive difference. **Statistically significant difference.

### Conditioned Pain Modulation

**Figure 3.**
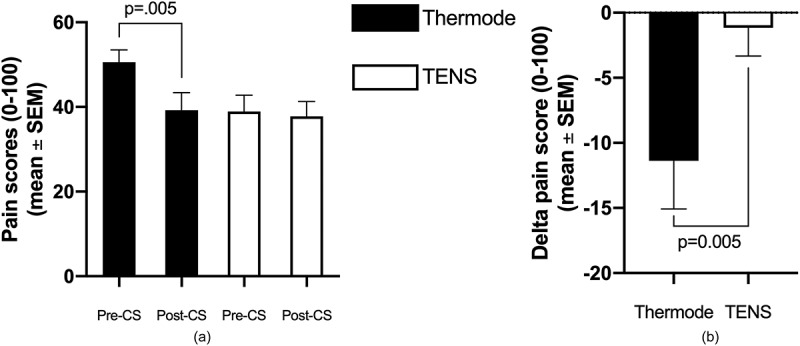
Average pain levels elicited by the thermode and TENS throughout the duration of the pre-CS TS. The linear regression (obtained from the pain scores at t=30, 60, 90 and 120 seconds) is shown for both paradigms. The slope of the linear regression represents temporal summation. Figure 3B shows the magnitude of CPM, calculated as the difference between post-CS and pre-CS pain scores

CPM, calculated by subtracting pre-CS TS pain score from post-CS TS pain scores, was larger following the thermode + CPTest than following the TENS (−11 ± 20/100 and −1 ± 12/100 respectively; *P* = 0.005; see Figure 3). There was no correlation (*r* = 0.29; *P* = 0.13) between CPM scores (continuous variable) induced by the two modalities. To assess the presence or absence of CPM induced by the CPTest and TENS for each participant, CPM was transformed from a continuous variable (delta scores) into a dichotomic variable (present/absent; see Methods). This allowed us to determine whether both paradigms (or only one, or neither) evoked CPM in each participant (Table 1). Twenty-four participants showed CPM with the thermode + CPTest paradigm compared to 14 with the TENS paradigm (*P* = 0.01). There was no association between participants’ responses to the two modalities (in other words, response to one modality was not associated with response to the other modality; φ = 0.08; *P* = 0.68). Table 2B summarizes CPM responses obtained with the two paradigms (thermode + CPTest vs. TENS).

## Discussion

The objective of this exploratory study was to determine whether TENS can be used to evaluate temporal summation and CPM in healthy participants. More specifically, we aimed to compare the average temporal summation and CPM scores obtained with the two paradigms to determine whether the two paradigms triggered these pain mechanisms in a similar number of participants, and whether response to one paradigm correlates with response to the other.

Our results showed that the average temporal summation evoked by the two paradigms was negligible (less than 0.1) and similar between the two paradigms. No significant correlation was found between individual temporal summation scores evoked by the two modalities (this negative result is unlikely to be due to lack of power, seeing as the correlation was weak). When the continuous temporal summation scores were transformed into dichotomic temporal summation present/absent results, the number of participants showing temporal summation during the pre-CS TS was similar in both paradigms (roughly two-thirds of participants). However, there was no association between response to the two modalities, such that response to one modality was not associated with response to the other.

As for CPM, the thermode + CPTest paradigm, but not the TENS paradigm, evoked an average response that was substantial (larger than 10 percentage points), and average CPM score was significantly larger with the thermode + CPTest compared to the TENS. No significant correlation was found between individual CPM scores evoked by the two modalities (again, this negative result is unlikely to be due to lack of power, seeing as the correlation was weak). When the continuous CPM scores were transformed into dichotomic CPM present/absent results, the number of participants showing CPM was larger in the thermode + CPTest paradigm compared to the TENS paradigm; in other words, the thermode + CPTest evoked CPM in a larger number of participants than the TENS. Moreover, there was no association between response to the two modalities (response to one modality was not associated with response to the other).

Together, these results suggest that the two paradigms are roughly equivalent in the ability to evoke temporal summation in participants (although response to one modality was not associated with response to the other) but that the TENS paradigm is less apt to induce CPM than the thermode + CPTest paradigm. It is somewhat surprising that the thermode + CPTest procedure did not evoke larger CPM and temporal summation responses on average, because a number of other studies have shown that thermodes and CPTest can in fact induce CPM^23,37,38^ and, to a lesser extent, temporal summation.^23,35,39^ One potential source of explanation may come from our study sample, which consisted mostly of university students, many of whom were familiar with the physiology of pain. It is possible that this knowledge led them to form expectations regarding their pain or otherwise influenced their pain perception. A recent study also observed that up to 14% of pain-free participants show no CPM hypoalgesia under experimental conditions.^40^ Should this lack of CPM effect be correlated with factors such as pain literacy, particular expectations or high levels of physical activity^36,41–43^ (factors that may have been disproportionately common among our participants given the homogeneity of our sample), our results may have been inordinately biased against CPM. Moreover, our TS consisted of a sustained, 120-s noxious heat stimulus generated by the thermode. However, in the literature, temporal summation is often assessed using a pulsed (as opposed to a continuous) stimulation.^25,44^ Some authors^25,45^ (but not all^46^) have proposed that a constant stimulation may actually be less effective than an intermittent stimulation in evoking temporal summation.

One major limitation in our study concerns the difference in pain intensity evoked by the two paradigms. Indeed, to compare the outcome of the two paradigms (temporal summation and CPM), the pre-CS TS and the CS should induce a similar pain intensity in both paradigms. However, on average, we observed that the pre-CS TS induced significantly more pain in the thermode paradigm (by 15 percentage). Moreover, the CS was also more painful (by 10 percentage points) in the thermode + CPTest paradigm compared to the TENS paradigm (the difference was not technically significant [*P* = 0.06] but is on the edge of the suggestivity threshold; though we cannot reject the null hypothesis and claim that there is a difference, we cannot claim that there is no difference either). As such, we cannot say whether differences in temporal summation/CPM reponse obtained from the two paradigms are attributable to these differences in pain levels or whether they arose from the differences in apparatus/modalities between the two paradigms.

Though TENS could be used instead of a thermode to measure temporal summation (but not CPM) in a clinical setting, readers should keep in mind that many of our participants responded only to one modality and not the other. These results are important because many studies assess temporal summation/CPM (for example, aiming to identify personal characteristics associated with the presence/absence of these mechanisms) using only a single modality.^18,23,38^ These studies tend to conclude that a certain subset of participants have deficient CPM or increased temporal summation. However, it may be that the mechanisms are not themselves deficient; they are simply less responsive to certain stimulations. Moreover, because some of our participants showed temporal summation in response to one modality but not the other, clinicians seeking to assess the presence of temporal summation in their participants in order to recommend a pharmacological treatment should choose their assessing modality carefully. Indeed, a stream of studies has started to assess the response of patients to certain classes of medication based on endogenous pain responses.^16,18,21^ Clinicians should therefore ensure that their clinical assessment of temporal summation is made using the same modality as the study they are basing their recommendations on.

## Conclusion

In conclusion, our results suggest that the two paradigms are roughly equivalent in the ability to evoke temporal summation in participants but that the TENS paradigm is less apt to induce CPM than the thermode + CPTest paradigm. Importantly, response to one modality did not predict response to another modality (especially in the case of temporal summation), such that conclusions regarding the presence/absence of endogenous pain mechanisms in certain individuals should be interpreted with caution and drawn preferably from studies using multiple modalities.
